# Natural Bone-Setting

**Published:** 1881-03

**Authors:** 


### NATURAL BONE-SETTING.

THE career of the Italian bone-setter, Regina Dal Cin, who has just returned to her native hills after a triumphal year of residence in this country, is like a bit of the surgery of a past age, revived from its sleep of a hundred years, to give us a demonstration of the manner in which surgery was divided into fragments, and illiterate specialists itinerated from place to place seeking occupation. The stone-cutting expeditions through Europe of Friar Jacques strongly resemble the bone-setting journeys of Madame Dal Cin to Vienna, Trieste and America. Even the record of the dirty monk that "most of the Parisians looked upon as a physician sent from heaven for the relief of mankind," is equalled by the claims for a special heaven-endowment made for the illiterate peasant woman. A native of an obscure village in the north of Italy, the daughter of an inn-keeper, without education, unable even to read in her own language, self-instructed, she has acquired such tact and facility in the manipulation of joints, which have been the subject of chronic diseases, that in selected cases she has been able to accomplish most excellent curative results. Her local reputation in Italy attracted the attention of an officer of high rank in in the U. S. Navy, who took his child, the subject as was believed of an incurable joint-disease, to her for treatment. The child having been greatly benefited, the case became the occasion of a highly wrought article descriptive of the powers of the *manipulatiste*, from the pen of the father, in one of the most popular magazines. A wealthy and influential family in Brooklyn, one of whose daughters was crippled from spinal and joint affections, attracted by her reputed powers, took their daughter to Italy and placed her under the care of this person, and eventually persuaded her to return to this country with them, where her stay was prolonged more than a year. Her arrival was heralded by long articles written by a clergyman, and published in the daily papers in which her supposed wonderful, mysterious, and God-given gifts of bone-setting were set forth with all the details which a vivid imagination and a good command of adjectives made possible. Large numbers of those who were halt and cripples from chronic hip and knee diseases were taken to her. As in the case of most others of those less pretentious natural bone-setters, who are found more or less thickly about the country, ignorant of anatomical knowledge and innocent of even the meaning of the word pathology, the invariably pathology of the cases brought to this woman, as she claimed was that a bone was "out," and that when by her manipulations the bone was restored to its place, a cure would be effected.

Her method of treatment was to keep the affected joint continuously poulticed from ten days to two weeks with a poultice of marsh-mallows; during this time gentle movements and firm rubbings were daily impressed upon the joint, and the expectation of the patient constantly excited as to the time when the final manœuvre was to be made by means of which the distorted bone was to be put back in its place. After sufficient of this preparatory treatment had been made, the final *coup de grace* was affected by a series of quick movements, and an exclamation from the operatress that at last it was done! As a rule, all her manipulations, even her final climacteric performances, were nearly painless. Great improvement in the usefulness of the affected joints resulted in many cases. This woman, now about sixty years of age, is said to be modest, kind and gentle in her manners, and is undoubtedly firmly and honestly persuaded that the conditions which she imagines to be present in her patients really exist; for how otherwise explain the success of her treatment, which has been so great as to secure for her, after repeated legal prosecution for practicing without qualifications, an official decree from the Minister of the Interior in the Kingdom of Italy, authorizing her to treat dislocations, fractures and hip disease!

A review of her methods shows that they are as perfect as if they had been devised by one profoundly versed in psychology and pathology. The expectation of the patient, his confidence in the result, and the co-operation of his will, are all enlisted by them. She probably never heard of Tuke on "The Influence of the Mind upon the Body," but she could not have done more wisely if she had closely studied it. She believes in herself as thoroughly as Bonaparte did in his "Star of Destiny;" to her every case is an engagement, and every patient she makes an Old Guard. The real condition of the joints, in the manipulation of which her success is so signal, can very rarely be that of dislocation or even of subluxation.

Those who have read that most delightful and profitable little book of Wharton P. Hood, entitled "On Bone-setting," will retain a vivid remembrance of the clear manner in which he demonstrates the function of persistent bands of adhesion, both extra and intra-articular, in producing permanent lameness of joints after all inflammation has subsided. The profession owes a debt of gratitude to Dr. Hood that he availed himself of the opportunity to study the methods of one of the ablest bone-setters, and that having pursued his studies after scientific methods he has given us the results as he has. These cases of lame joints confront every practitioner continually; too often they constitute an *opprobrium chirurgicum:* and when, after having been long unrelieved through the want of skill or attention of the educated practitioner, they are cured by the manipulations of a bone-setter, they bring scientific surgery into discredit. It is our conviction that in a majority of cases of stiff joints the obstacle to motion exists less in intra-articular effusions and adhesions than it does in the changes which the extra-articulate structures have undergone; stiffened and adherent muscles and ligaments, contracted fasciæ, tendons and sheaths inseparably blended, conditions which have developed from old inflammations about a joint or from prolonged disuse after bruises or other injuries, these are the chief causes of disability. How admirably the emollient poultices, the rubbings and the movings practiced by the Italian woman, are adapted to promote the absorption of effusions, the stretching and rupture of adhesions, and the restoration of tone to debilitated structures! It is true that no new principle, no new method, is found in her practice. She is but a skillful *masseuse.* The reason why physicians in general so often fail to relieve the cases in question, and so permit them to fall into the hands of empirics, is that these details of poulticing and rubbing and moving demand a degree of patience and devotion to trifling details which is possessed by few. As in so many other things the brilliant successes of such a person as Madame Dal Cin alone are heard of, while of the many failures and occasional disasters nothing is said. We are cognizant of one case in which she awakened in a hip-joint acute inflammation which terminated in abscess and necrosis. The scientific surgeon weighing carefully all the elements of the problem, which each case that comes before him presents, will determine what cases are the proper ones to submit to manipulation and what to leave undisturbed. In his hands the same brilliant successes will be secured by the same methods in the one class of cases, while in the other disasters will be prevented. Under the guidance of the educated surgeon, Madame Dal Cin and such as she might do work of yet greater value. The methods of the natural bone-setter, as in the case of the English Hutton and the Italian Dal Cin, may sometimes repay the consideration of the educated surgeon. It may be a mistake to always ignore them, or to invariably dismiss their work with a sneer and the epithet of quackery.—*Annals of Anat. and Surg.*, Jan., 1881.

